# Effect of Monocot Grass Extract (MGE) on mood state and sleep patterns in moderately stress subjects

**DOI:** 10.1186/1550-2783-10-S1-P26

**Published:** 2013-12-06

**Authors:** Shawn M Talbott, Julie A Talbott

**Affiliations:** 1 SupplementWatch, Draper, UT 84020 USA

## Background

Overtraining syndrome (OTS) is a stress-related phenomenon experienced by elite-level and recreational athletes alike. Athletes are subjected to stressors from physical, psychological, and biochemical sources that may lead to OTS and significant decrements in mental and physical performance. OTS may be characterized by elevated perceived stress, reduced mood quality, increased tension/anxiety, and disrupted sleep quality/quantity; each of which can influence and compound the other, leading to a vicious cycle of increasingly poor performance, increased stress, and disrupted sleep patterns.

## Methods

In this study, we supplemented moderately stressed subjects with an extract of monocot grasses (corn grass, wheat grass, and bamboo). Previous animal studies have shown significant anti-stress and relaxation benefits of monocot grass extracts (MGE), likely due to their content of plant metabolite 6-MBOA (6-methoxybenzoxazolinone) and its ability to influence serotonin levels. Fifty-two subjects were randomly assigned in double-blind fashion to receive MGE (N=27, 18 Female & 9 Male) or Placebo (N=25, 17 Female & 8 Male) for 4 weeks. We measured Mood State (Profile of Mood States), Sleep Quality (Pittsburgh Sleep Quality Index), and Sleep Patterns (ZEO Sleep Monitor) before and after 4 weeks of supplementation. Differences between MGE/Placebo at week 4 were analyzed by paired t-tests with an alpha level of 0.05 and reported as percent-difference between groups.

## Results

Compared to the Placebo group, the MGE group (all p < 0.05):

• Had 8% less Tension (7.9 + 5.9 v. 8.6 + 5.5)

• Had 15% less Depression (6.8 + 6.9 v. 8.0 + 7.9)

• Had 25% less Irritability (6.4 + 5.0 v. 8.0 + 7.9)

• Fell asleep 33% faster (0.63 + 0.79 v. 0.84 + 0.90)

• Had 50% better sleep "efficiency" (0.26 + 0.59 v. 0.52 + 0.71)

• Had 40% better sleep "quality" (0.67 + 0.48 v. 1.12 + 0.97)

• Woke up 30% fewer times each night (2.1 + 2.5 v. 3.0 + 1.5)

• Experienced 24% more time in deep REM sleep (1.85 + 0.46h v. 1.41 + 0.30h)

## Conclusion

Overall, these results indicate that the MGE supplement is effective in improving sleep quality and improving stress-related mood states in a population of moderately stressed subjects. Future studies are warranted to evaluate the specific effects of MGE in alleviating OTS in athletes and possibly improving physical and mental performance.

